# A Word of Caution in the Administration of Acid Medicines

**Published:** 1880-04

**Authors:** 


A Word of Caution in the Administration of Acid Medicines.—It was with much interest that I read the article on the "Insufficient Formation of Gastric Juice" in the January number of The Physician and Surgeon. The course of treatment being the best, it was to be expected that recovery would result, but I noticed what I consider an important omission in the instructions to the patient. It was ordered that the acid prescription should be taken through a glass tube. This is all right, but it is not enough, the patient should have been further ordered to rinse the mouth after the use of the acid with a solution of bicarbonate of soda. The reason is obvious: for no pains should be spared to protect the teeth from the action of strong acids. A neglect of this caution by physicians has probably caused many dyspeptics. By observing, you will find that many of the patients who present themselves for treatment for dyspepsia and its kindred diseases are wanting in efficient masticatory apparatus. Either on account of decayed teeth or from numerous extractions, mastication is performed in the one case with pain and in the other very inefficiently. What is the result of such a condition of the teeth? These persons resort to food that needs little or no chewing, and which consequently calls for but little exertion on the part of the stomach. Lack of exercise is but the prelude to lack of secretion. This eventually brings about a train of disorders, which could easily be controlled in their incipiency, but which, allowed to go on, often prove to be most formidable cases.

That a great many teeth are lost by the action of strong medicines cannot be denied. I have now two patients, sisters, whose teeth are almost ruined through the use of the tincture of the chloride of iron. They had been taking the medicine through tubes, but I suggested the wash of solution of bicarbonate of soda, and the teeth have been greatly improved, and relieved of that bluish cast which teeth have when they are subjected to the action of acids.—*The Physician and Surgeon.*

